# Perceived stress and smoking across 41 countries: A global perspective across Europe, Africa, Asia and the Americas

**DOI:** 10.1038/s41598-017-07579-w

**Published:** 2017-08-08

**Authors:** Brendon Stubbs, Nicola Veronese, Davy Vancampfort, A. Mathew Prina, Pao-Yen Lin, Ping-Tao Tseng, Evangelos Evangelou, Marco Solmi, Cristiano Kohler, André F. Carvalho, Ai Koyanagi

**Affiliations:** 1 Physiotherapy Department, South London and Maudsley NHS Foundation Trust, Denmark Hill, London, SE5 8AZ United Kingdom; 2 Health Service and Population Research Department, Institute of Psychiatry, Psychology and Neuroscience, King’s College London, De Crespigny Park, London, Box SE5 8AF United Kingdom; 3 Faculty of Health, Social Care and Education, Anglia Ruskin University, Chelmsford, United Kingdom; 4 Institute for Clinical Research and Education in Medicine, I.R.E.M., Padova, Italy; 5 KU Leuven Department of Rehabilitation Sciences, Leuven, Belgium; 6 KU Leuven, University Psychiatric Center KU Leuven, Leuven-Kortenberg, Belgium; 7 Department of Psychiatry, Kaohsiung Chang Gung Memorial Hospital and Chang Gung University College of Medicine, Kaohsiung, Taiwan; 8 Institute for Translational Research in Biomedical Sciences, Kaohsiung Chang Gung Memorial Hospital, Kaohsiung City, Taiwan; 9 Department of Psychiatry, Tsyr-Huey Mental Hospital, Kaohsiung Jen-Ai’s Home, Taiwan; 10 Department of Hygiene and Epidemiology, University of Ioannina Medical School, Ioannina, Greece; 11 Department of Biostatistics and Epidemiology, Imperial College London, London, UK; 12 Department of Neurosciences, University of Padova, Padova, Italy; 13 Local Health Unit 17 ULSS 17, Mental Health Department, Padova, Italy; 14 Department of Clinical Medicine and Translational Psychiatry Research Group, Faculty of Medicine, Federal University of Ceará, Fortaleza, CE Brazil; 15 Research and Development Unit, Parc Sanitari Sant Joan de Déu, Universitat de Barcelona, Fundació Sant Joan de Déu, Dr. Antoni Pujadas, 42, Sant Boi de Llobregat, Barcelona, 08830 Spain; 16 Instituto de Salud Carlos III, Centro de Investigación Biomédica en Red de Salud Mental, CIBERSAM, Monforte de Lemos 3-5 Pabellón 11, Madrid, 28029 Spain

## Abstract

Within recent years, there has been a seismic shift in smoking rates from high-income to low- and middle-income countries (LMICs). Evidence indicates that perceived stress may comprise a barrier for smoking cessation, but little is known about the association of perceived stress and smoking in LMICs. We conducted a cross-sectional, community-based study comprising 217,561 people [mean age 38.5 (SD = 16.1) years, 49.4% males]. A perceived stress score [range 2 (lowest-stress) 10 (highest-stress)] was computed from the Perceived Stress Scale. Multivariable logistic regression analyses were conducted. In the overall sample, a one-unit increase in perceived-stress resulted in a 5% increased odds of smoking (OR = 1.05; 95%CI = 1.03–1.06). Increased stress was associated with smoking in Africa (OR = 1.06; 95%CI = 1.04–1.09), Americas (OR = 1.03; 95%CI = 1.01–1.05), and Asia (OR = 1.06; 95%CI = 1.04–1.08), but not Europe (OR = 0.99; 95%CI = 0.95–1.02). Increasing levels of perceived stress were significantly associated with heavy smoking (≥30 cigarettes per day) among daily smokers (OR = 1.08; 95%CI = 1.02–1.15). A country-wide meta-analysis showed that perceived stress is associated with daily smoking in most countries. Prospective studies are warranted to confirm/refute this relationship, which may have meaningful public health implications.

## Introduction

Tobacco use is a significant global public health issue and there are approximately 1 billion smokers in the world, with 80% of those currently living in low- and middle-income countries (LMICs)^1^. There is a burgeoning evidence base that tobacco use is a leading modifiable contributor to global mortality, and approximately half of smokers will experience premature death due to cardiovascular, respiratory, neoplastic or other associated diseases^2–4^. Understanding the factors that may be independently associated with tobacco use is therefore a global public health priority.

There is a growing body of evidence suggesting that high levels of perceived stress are associated with increased prevalence of smoking^5–7^. Perceived stress can be defined as feelings or thoughts that an individual has about how much stress they are under, as well as feelings about the uncontrollability and unpredictability of one’s life, how often one has to deal with irritating hassles, how much change is occurring in one’s life, and confidence in one’s ability to deal with problems or difficulties^8^. A recent systematic review found that 40 qualitative studies endorsed that smoking is a major strategy to ‘manage stress’ while it decreases arousal levels^9^. Whilst there have been concerns that stopping smoking may result in increased arousal and stress levels and consequently a deterioration of mental health^10^, a recent meta-analysis found that anxiety, depression and stress all significantly decreased after smoking cessation^11^.

To date, the majority of research considering the relationship between smoking and perceived stress has focused on high-income countries. However, recent research has demonstrated that as the tobacco epidemic is being tackled in high-income countries, the prevalence and burden of smoking is rapidly spreading to LMICs^12–16^. There is a rapid increase in non-communicable diseases in LMICs, at least in part explained by changes in lifestyles in these countries^17, 18^. Relatively little information about correlates of smoking is known in LMICs and understanding such information is a potentially important factor for public health interventions^19^. In LMICs, perceived stress levels are also known to be significantly increased compared to developed nations^20^, yet nationally representative multi-national data exploring the relationship between -perceived stress and smoking in LMICs are to the best of our knowledge lacking. There is also a lack of clarity on whether or not the relationship between perceived stress and smoking is different across geographical regions.

Given the above that (1) smoking is a leading cause of global preventable death^2–4^, (2) recent research has demonstrated high perceived stress is associated with high rates of smoking in Western countries^5–7^, (3) understanding important correlates/barriers to smoking cessation such as perceived stress are a global public health priority and (4) there is an absence of multi-national research considering perceived stress and smoking behaviours in LMICs (where approximately 80% of smokers reside), the aims of the current study were to assess the relationship between perceived stress and smoking or heavy smoking using community-based data from 41 countries (predominantly LMICs) which provided data to the World Health Survey (WHS).

## Data and Methods

The current study used data from the World Health Survey (WHS), a cross-sectional, community-based study undertaken in 2002–2004 in 70 countries worldwide. Single-stage random sampling and stratified multi-stage random cluster sampling were conducted in 10 and 60 countries respectively. Full details of the survey are available elsewhere (http://www.who.int/healthinfo/survey/en/). Briefly, people who were aged 18 years and older with a valid home address were eligible to participate. Kish tables were used to ensure that each member of the household had equal probability of being selected. The data were collected in all countries using the same questionnaire, although some countries used an abridged version. The individual response rate (i.e. the ratio of completed interviews among selected respondents after excluding ineligible respondents from the denominator) ranged from 63% (Israel) to 99% (Philippines)^21^. The WHS received ethical approval from the ethical boards at each study site (Appendix 1). Sampling weights were generated to adjust for non-response and the population distribution reported by the United Nations Statistical Division. Written informed consent was obtained from all participants. All methods were performed in accordance with the relevant guidelines and ethical approval obtained.

### Variables

#### Current smoking and heavy smoking (outcome variables)

The question ‘Do you currently smoke any tobacco products such as cigarettes, cigars, or pipes’? with the answer options being ‘daily’, ‘yes, but not daily’, or ‘no, not at all’ was used to identify current smokers. Those who replied ‘daily’ or ‘yes, but not daily’ were considered to be smokers. A follow-up question on the average daily consumption of each tobacco products (manufactured cigarettes, hand-rolled cigarettes, pipefuls of tobacco, and other) was asked to those who smoked ‘daily’. We calculated the total number of all types of cigarettes and other forms of tobacco smoked daily. Individuals who smoked ≥ 30 cigarettes or other tobacco products per day were coded as heavy smokers^22^.

### Perceived stress (exposure variable)

Perceived stress was captured in accordance with previous WHS publication^23^, over the last month utilizing two questions from the Perceived Stress Scale^24^. The questions used were (1) “How often have you felt that you were unable to control the important things in your life”?; and (2) “How often have you found that you could not cope with all the things that you had to do”? Respondents answered to these questions as: never (score = 1), almost never (score = 2), sometimes (score = 3), fairly often (score = 4), very often (score = 5). The scores of the two questions were added to create a scale ranging from 2 to 10 with greater scores indicating a higher level of perceived stress^23^. The overall correlation coefficient between these 2 questions was 0.74 in the final sample including 41 countries.

### Other variables

Data on sex, age (18–34, 35–59, ≥60 years), wealth quintiles, and setting (rural or urban) were used as control variables. Country-wise wealth quintiles were created using principal component analysis based on 15–20 assets including country-specific items for some countries. The selection of those control variables was based on past literature^25^.

### Statistical analysis

Data were publically available for 69 countries. The data were nationally representative for all countries with the exception of China, Comoros, the Republic of Congo, Ivory Coast, India, and Russia. Countries without any sampling information (10 countries – Austria, Belgium, Denmark, Germany, Greece, Guatemala, Italy, Netherlands, Slovenia, UK) were excluded. Twelve countries (Brazil, Finland, France, Hungary, Ireland, Israel, Luxembourg, Norway, Portugal, Sweden, Turkey, Zimbabwe) were also omitted, as data on smoking/perceived stress were not collected. In addition, Georgia was excluded owing to a negative correlation between the two questions on perceived stress^23^. Finally, we omitted five further countries (Mali, Ecuador, Slovakia, Congo, Swaziland) as more than 25% of data on smoking/perceived stress was missing. Thus, the final analytical sample consisted of 217,561 people from a total of 41 countries. According to the United Nations’ classification system (http://unstats.un.org/unsd/methods/m49/m49regin.htm), this corresponded to 16 countries in Africa (n = 67,056), 4 in the Americas (n = 52,057), 13 in Asia (n = 79,866), and 8 in Europe (n = 18,562). Furthermore, according to the World Bank classification at the time of the survey (2003), these countries corresponded to 2 high-income (n = 7556), 21 middle-income (n = 116,970), and 18 low-income (n = 93,035) countries. The exact countries included and their regions and sample size are provided in the Appendix 2.

The difference in the prevalence of smoking by sample characteristics was tested by Chi-squared tests. Multivariable logistic regression analysis was conducted to assess the association between perceived stress (exposure variable) and smoking (outcome variable). We also conducted analyses stratified by sex as previous research has shown that the association between smoking and perceived stress may differ by sex^25^. The analyses were conducted using the overall sample including all 41 countries and also by region. Analyses were also conducted by middle-income and low-income countries. Analyses only on high-income countries were not done as there were only two high-income countries (United Arab Emirates and Spain) and we judged that the results are unlikely to be representative of this context. We also assessed whether perceived stress is associated with heavy smoking among daily smokers by conducting multivariable logistic regression analyses with stress as the exposure and heavy smoking as the outcome restricting to daily smokers. We only conducted this analysis using the overall sample as the number of heavy smokers was small (n = 1567). In addition, we also assessed whether levels of perceived stress differ among daily smokers and non-daily smokers using the overall sample. All the above-mentioned models were adjusted for sex, age, wealth, setting, and country, with the exception of the sex-wise analyses which were not adjusted for sex. As in previous WHS publications, country was adjusted for by including dummy variables for each country^23, 26^. Next, country-wise multivariable logistic regression models were constructed to assess the association between perceived stress and smoking, adjusting for age, sex, wealth, and setting. The estimates for each country were also combined into a random-effect meta-analysis with the Higgins’ I^2^ statistic being calculated. The Higgins’ I^2^ represents the degree of heterogeneity between countries that is not explained by sampling error with a value <40% often considered as negligible and 40–60% as moderate heterogeneity^27^.

Under 5% of the values were missing for all the variables used in the analysis with the exception of wealth (7.6%). For all regression analyses, the covariates were included in the models as categorical variables apart from the perceived stress scale (continuous variable), and complete-case analysis was done. The sample weighting and the complex study design were taken into account in all analyses with Taylor linearization methods. Results from the logistic regression models are presented as ORs with 95% confidence intervals (95%CIs). The level of statistical significance was set at P < 0.050. All statistical analyses were performed with the Stata statistical software version 14.1 for Windows (Stata Corp LP, College Station, Texas).

## Results

**Figure 1:**
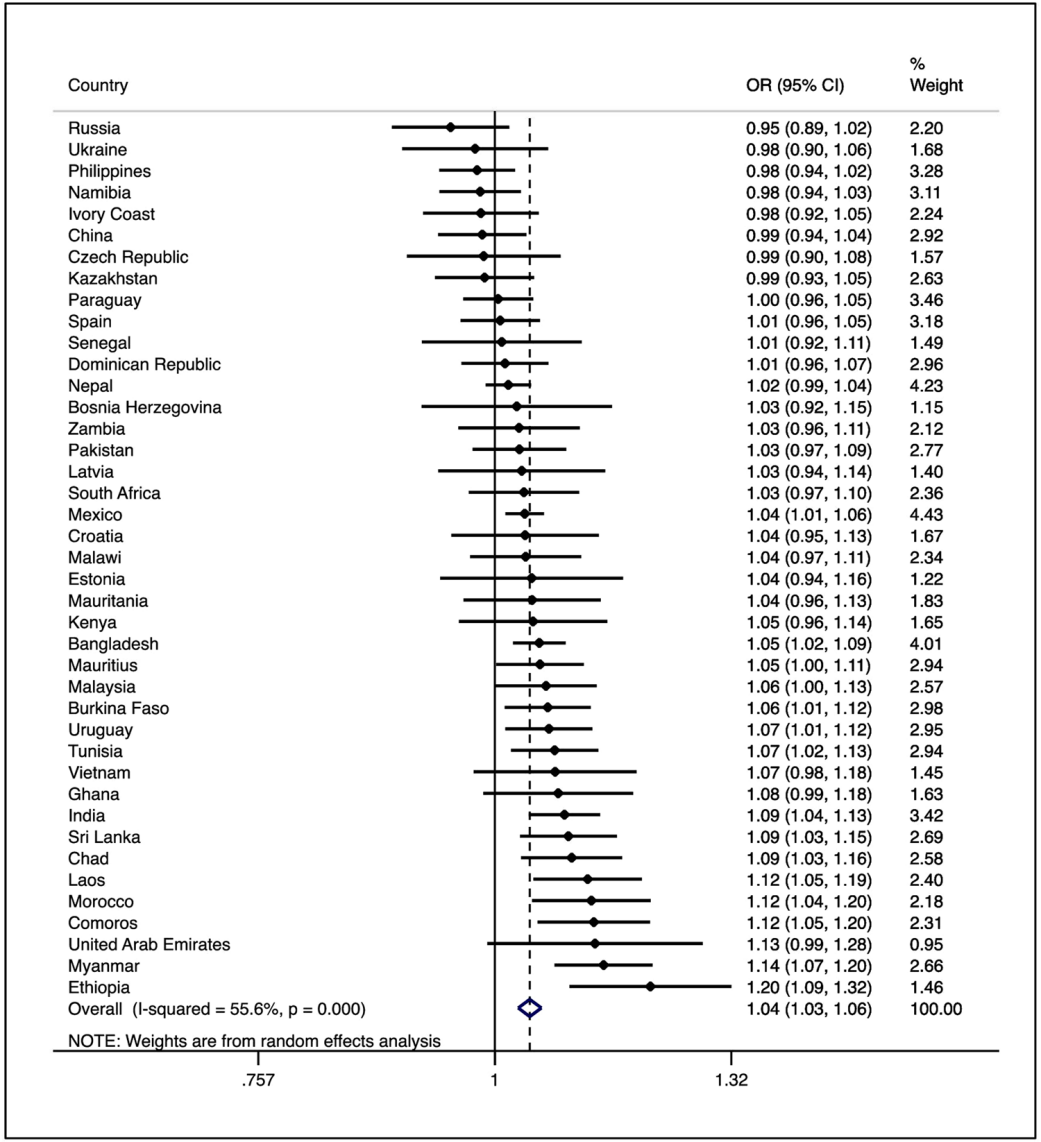
Country-wise association between perceived stress and smoking (outcome) estimated by multivariate logistic regression Abbreviation: OR odds ratio; CI confidence interval Models are adjusted for age, wealth, and setting. The perceived stress score ranged from 2–10 with higher scores corresponding to higher levels of stress.

The average age (SD) of the overall sample was 38.5 (16.1) years and 49.4% were males. The overall and region-wise sample characteristics are shown in Table 1. Overall, the prevalence of smoking was 27.3% with the lowest and highest prevalence being observed in Africa (13.4%) and Asia (32.1%) respectively. There was a much larger proportion of older (≥60 years) individuals in Europe (29.8%) compared to Africa (9.6%). The mean perceived stress score ranged from 3.7 in the Americas to 5.1 in Africa. The prevalence of smoking by sample characteristic is provided in Table 2. With the exception of males being more likely to smoke in all continents, there were distinct patterns for other characteristics by continent. For example, older individuals were much less likely to smoke in Europe. The association between perceived stress and smoking estimated by multivariable logistic regression is presented in Table 3. In the overall sample, a one-unit increase in the perceived stress scale (range 2–10) was associated with a 1.05 (95%CI = 1.03–1.06) times higher odds for smoking. Similar results were found in Africa, the Americas, and Asia but there were no significant associations observed in Europe (OR = 0.99; 95%CI = 0.95–1.02). In order to assess whether the association in Europe is significantly different from other continents, we included a product term “Europe (Y/N) X perceived stress” in the model using the overall sample. The results showed that the difference is statistically significant (P < 0.0001). The association of perceived stress with smoking in low-income and middle-income countries were similar (Appendix 3). In the sex-stratified analyses, similar results were found for both males and females (Table 4). Although the association was only significant among males in Africa, or among females in the Americas, when we tested for interaction by including the product term “sex X perceived stress” in the models, this difference was not statistically significant. In the country-wise analyses, the OR (95%CI) associated with a one-unit increase in the perceived stress scale for smoking ranged from 0.95 (0.89–1.02) in Russia to 1.20 (1.09–1.32) in Ethiopia (Fig. 1). The overall OR (95%CI) based on a meta-analysis was 1.04 (1.03–1.06) with a moderate level of heterogeneity being observed (I^2^ = 55.6%). Finally, the OR of perceived stress for heavy smoking among daily smokers was 1.08 (95%CI = 1.02–1.15; p = 0.008), and there were no significant differences in the levels of perceived stress between non-daily and daily smokers (data not shown).

**Table 1:** Sample characteristics (overall and by region).

Characteristic	Category	Overall		Africa		Americas		Asia		Europe	
		N^a^		N^a^		N^a^		N^a^		N^a^	
Smoking	No	158,723	72.7	53,922	86.6	39,021	75.2	52,780	67.9	13,000	69.3
	Yes	51,601	27.3	9,655	13.4	12,330	24.8	24,193	32.1	5,423	30.7
Sex	Female	117,209	50.6	35,071	51.1	29,118	51.7	41,691	49.0	11,329	57.1
	Male	94,427	49.4	29,317	48.9	22,284	48.3	35,699	51.0	7,127	42.9
Age (years)	18–34	88,993	47.8	31,112	55.0	21,614	48.5	32,099	49.2	4,168	26.8
	35–59	89,356	38.9	24,848	35.4	21,134	39.3	35,600	39.3	7,774	43.4
	≥60	33,206	13.3	8,359	9.6	8,652	12.2	9,687	11.6	6,508	29.8
Wealth	Poorest	47,938	20.1	14,173	20.1	11,862	20.0	17,543	20.1	4,360	20.0
	Poorer	42,241	20.0	12,410	20.3	11,334	20.0	14,870	19.9	3,627	20.0
	Middle	38,891	19.9	11,218	19.7	10,301	20.0	14,171	20.0	3,201	20.0
	Richer	36,858	20.0	10,995	20.0	9,359	20.0	13,452	20.0	3,052	20.0
	Richest	34,761	20.0	11,142	20.0	8,038	20.0	12,790	20.0	2,791	20.0
Setting	Rural	109,903	60.3	39,408	61.9	14,770	26.5	51,126	71.4	4,599	24.0
	Urban	106,467	39.7	26,821	38.1	37,277	73.5	28,656	28.6	13,713	76.0
Perceived stress score	Mean (SD)	208,539	4.8 (2.2)	62,674	5.1 (2.2)	51,277	3.7 (1.8)	76,478	4.9 (2.2)	18,109	4.5 (2.0)

^a^Unweighted N.

Data are percentage unless otherwise stated. Percentage and mean (SD) are based on weighted sample.

^b^The perceived stress score ranged from 2–10 with higher scores corresponding to higher levels of perceived stress.

**Table 2:** Prevalence of smoking by sample characteristics.

	Overall		Africa		Americas		Asia		Europe	
	%	P-value	%	P-value	%	P-value	%	P-value	%	P-value
**Age** (years)	Yes		Yes		Yes		Yes		Yes	
18–34	21.8	<0.0001	10.8	<0.0001	23.6	0.0001	24.0	<0.0001	40.7	<0.0001
35–59	33.9		17.4		26.3		40.0		36.2	
≥60	27.8		13.5		24.6		39.9		13.6	
**Sex**										
Female	12.4	<0.0001	4.1	<0.0001	15.3	<0.0001	14.4	<0.0001	16.0	<0.0001
Male	42.5		23.2		34.9		49.1		50.1	
**Wealth**										
Poorest	31.2	<0.0001	15.7	<0.0001	21.0	<0.0001	39.6	<0.0001	25.1	<0.0001
Poorer	29.1		14.1		23.1		35.8		28.6	
Middle	28.0		13.5		25.4		32.6		33.1	
Richer	26.1		13.1		25.3		29.3		35.1	
Richest	22.1		10.8		29.2		23.1		31.9	
**Setting**										
Rural	28.5	<0.0001	11.7	<0.0001	19.3	<0.0001	34.2	<0.0001	29.8	0.4544
Urban	25.6		16.2		26.7		26.8		30.9	

Percentages are based on weighted sample.

The difference in the prevalence of smoking by sample characteristics was tested by Chi-squared tests.

**Table 3:** Association between perceived stress and smoking (outcome) estimated by multivariable logistic regression.

	Overall			Africa		P-value	Americas		P-value	Asia		P-value	Europe		P-value
	OR	95%CI	P-value	OR	95%CI		OR	95%CI		OR	95%CI		OR	95%CI	
Perceived stress^a^	1.05	(1.03–1.06)	<0.0001	1.06	(1.04–1.09)	<0.0001	1.03	(1.01–1.05)	0.0011	1.06	(1.04–1.08)	<0.0001	0.99	(0.95–1.02)	0.3863
**Age** (years)															
18–34	1.00			1.00			1.00			1.00			1.00		
35–59	2.02	(1.92–2.13)	<0.0001	1.85	(1.65–2.07)	<0.0001	1.17	(1.09–1.26)	<0.0001	2.53	(2.35–2.72)	<0.0001	0.82	(0.71–0.95)	0.0068
≥60	1.42	(1.30–1.55)	<0.0001	1.30	(1.11–1.53)	0.0014	1.07	(0.95–1.20)	0.2631	2.59	(2.29–2.93)	<0.0001	0.22	(0.18–0.27)	<0.0001
**Sex**															
Female	1.00			1.00			1.00			1.00			1.00		
Male	6.37	(6.00–6.76)	<0.0001	8.72	(7.64–9.94)	<0.0001	3.14	(2.91–3.39)	<0.0001	7.48	(6.87–8.13)	<0.0001	5.32	(4.64–6.10)	<0.0001
**Wealth**															
Poorest	1.00			1.00			1.00			1.00			1.00		
Poorer	0.86	(0.80–0.92)	<0.0001	0.85	(0.73–0.99)	0.0329	1.05	(0.94–1.18)	0.3543	0.79	(0.72–0.88)	<0.0001	0.87	(0.73–1.03)	0.1042
Middle	0.80	(0.74–0.87)	<0.0001	0.82	(0.70–0.95)	0.0085	1.18	(1.05–1.32)	0.0048	0.68	(0.60–0.76)	<0.0001	0.83	(0.69–1.00)	0.0561
Richer	0.70	(0.64–0.77)	<0.0001	0.73	(0.61–0.86)	0.0002	1.12	(1.00–1.26)	0.0597	0.56	(0.50–0.64)	<0.0001	0.81	(0.66–0.98)	0.0324
Richest	0.52	(0.47–0.56)	<0.0001	0.54	(0.45–0.65)	<0.0001	1.37	(1.21–1.55)	<0.0001	0.36	(0.32–0.41)	<0.0001	0.65	(0.53–0.80)	0.0001
**Setting**															
Rural	1.00			1.00			1.00			1.00			1.00		
Urban	1.05	(0.98–1.13)	0.1703	1.21	(1.04–1.41)	0.0132	1.45	(1.31–1.61)	<0.0001	0.95	(0.85–1.06)	0.3593	1.23	(1.04–1.44)	0.0132

Abbreviation: OR odds ratio; CI confidence interval.

^a^The perceived stress score ranged from 2–10 with higher scores corresponding to higher levels of perceived stress.

Models are adjusted for age, sex, wealth, setting, and country.

**Table 4:** Sex-stratified association between perceived stress and smoking (outcome) estimated by multivariable logistic regression.

	Male	95%CI	P-value	Female	95%CI	P-value
	OR			OR		
Overall	1.05	(1.03–1.06)	<0.0001	1.05	(1.03–1.07)	<0.0001
Africa	1.07	(1.04–1.10)	<0.0001	1.05	(1.00–1.10)	0.0590
Americas	1.02	(0.99–1.04)	0.1975	1.06	(1.03–1.09)	0.0001
Asia	1.05	(1.03–1.08)	<0.0001	1.05	(1.02–1.08)	0.0039
Europe	0.97	(0.93–1.02)	0.1999	1.02	(0.97–1.06)	0.5016

Abbreviation: OR odds ratio; CI confidence interval

The perceived stress score ranged from 2–10 with higher scores corresponding to higher levels of perceived stress.

Models are adjusted for age, wealth, setting, and count.

## Discussion

To the best of our knowledge, our study is the largest of its kind and contains several novel results. First, our data suggest that perceived stress is significantly associated with higher smoking rates: each one-unit increase in the perceived stress scale (range 2–10) was associated with a 1.05 times higher odds for smoking in the pooled sample, while a moderate level of heterogeneity for this association was observed in country-wise analyses. Second, our study showed that this association was significant in Africa, the Americas, and Asia, but not in Europe. Finally, among daily smokers, higher levels of self-perceived stress were associated with increased odds for heavy smoking.

Surprisingly, very little information is available concerning the relationship between perceived stress and smoking behaviours among LMICs and virtually all of our understanding of this relationship is derived from high-income countries. To the best of our knowledge, our study is the first multi-national study to consider the relationship between perceived stress and smoking across a large number of LMICs. A previous study among Latinos and Hispanic ethnic minorities in the United States (N = 5,313) found that perceived stress was associated with increased odds for smoking (OR = 1.03; 95%CI = 1.01–1.05)^28^. Other research among 263 low income African American women in the United States also identified that higher perceived stress was associated with higher levels of smoking and closely linked to alcohol use in ref. 29, while a representative sample of 263 Puerto Rican college students identified that smoking was widely reported as being a strategy used to deal with academic stress^30^. Furthermore, a study among 1,595 people in China that found migrants with higher perceived life stress were more likely (OR = 1.45; 95%CI = 1.05–2.06) than migrants with lower levels of stress to be current smokers^31^. A similar relationship was observed in this study when considering perceived work stress in migrants also. Finally, another study in China across 7 cities, including 4,072 male smokers identified that lower perceived stress was associated with a higher odds of being abstinent from smoking^32^.

Several theories have been proposed to explain the association between perceived stress and cigarette smoking, although to the best of our knowledge, these are all set in the context of high-income countries. Perhaps the most prominent hypothesis is that stress may increase hypothalamus-pituitary-adrenal (HPA) axis reactivity, negative emotions, physiologic reactivity and therefore craving for nicotine^33^. Nicotine is known to have an acute impact on the HPA axis, yet, chronic nicotine exposure appears to dysregulate the HPA axis^34–36^. Experimentally induced stress has identified that stress reduces the ability of people to resist cigarette smoking and people who smoke under stressful situations get increased reward (in the short term) from doing so ref. 33. Another interpretation is that smoking is used as a strategy to relieve the stress of daily life in LMICs. Specifically, smokers with greater perceived stress experience greater negative reinforcement smoking expectations, which in turn, may be related to numerous processes involved in the maintenance of smoking^37^. Perceived stress may constitute a barrier for the prevention of smoking initiation and also for the achievement of smoking cessation. Focus group research among low-income people in New York suggested that perceived stress reinforces unhealthy behaviours including smoking, which in turn act as a short term solution to aid in the ‘management’ of their stress^38^. However, despite concerns of a potential deterioration in stress, studies have found that even among highly dependent smokers, stopping smoking is not associated with any long term increases in perceived stress^11^. Thus, perceived stress need not be a precluding barrier to commencing smoking cessation, although it may be important to address the issue of perceived stress to help people stop smoking. However, it is noteworthy, that the association between perceived stress and smoking may not be homogeneous across countries and regions, and future research is required to explore factors associated with the differences herein observed.

Tobacco prevention and control strategies have a strong scientific basis, yet a distinct gap remains between this evidence and implementation of tobacco control policies, particularly in LMICs. Although policy strengthening had been conducted in the last decade, room for considerable improvement remains, particularly in LMICs^19^. Taken together with the wider literature, our data suggest that perceived stress is closely related to smoking rates in LMICs. In order to be successful, interventions that target smoking cessation and concerns regarding stress might prove useful, although there is sparse data to confirm/refute this in the context of LMICs. Health professionals can contribute to tobacco control efforts, especially through patient-level clinical interventions, but only when supported by a health care system and government that recognize and support tobacco control as a critical strategy for health promotion and chronic disease prevention. Tobacco prevention and control should be a task for health professionals at all levels of care worldwide^39^. Our data show that tobacco prevention and control programs should include an assessment of perceived stress and education about healthy coping strategies (e.g. breathing exercises). A recent study in India showed that single session quit advice (15 min) plus a single training session in yogic breathing exercises can increase tobacco cessation, even in a LMIC setting^40^.

Some study design limitations need to be considered. First of all, due to the cross-sectional design of the study, causality or temporal associations cannot be established. Given the wider literature on perceived stress and smoking, it is likely that a bidirectional relationship exists. Second, perceived stress was recorded through a self-reported measure and may be subject to recall bias. Similarly, it is possible that some people reported less smoking rates than true for social desirability. Third, only two high-income countries (United Arab Emirates and Spain) were included in the analysis. Future studies with more high-income countries are warranted in order to obtain a more global understanding of the association between perceived stress and smoking. Fourth, potential confounding variables may mediate the association between perceived stress and smoking. For example, a recent cross-sectional survey conducted in China found that anxiety and depression may mediate the relationship between stress and smoking. Future longitudinal studies are needed to elucidate possible moderators/mediators as well as mechanisms of the relationships we observed.

In conclusion, our data show that perceived stress is significantly associated with higher smoking rates across LMICs. There was some evidence of relevant differences across geographical regions and future research should attempt to explore potential reasons for this. Clearly, prospective research is required to determine the directionality of the relationships we observed, while preventive strategies and the possible treatments targeting perceived stress may be promising directions for tackling the smoking epidemic in LMICs.

## Electronic supplementary material

Supplementary Information
