# Supplementary material for: Perceived stress and smoking across 41 countries: A global perspective across Europe, Africa, Asia and the Americas
Source: Sci Rep. 2017 Aug 8;7:7597. doi: 10.1038/s41598-017-07579-w (PMC5548752; doi:10.1038/s41598-017-07579-w)
Supplement: Supplementary file 1 — Supplementary Information [file 41598_2017_7579_MOESM1_ESM.pdf]

Perceived stress and smoking across 41 countries: A global perspective across Europe, Africa, Asia and the Americas

Running title: stress and smoking

Submission to Scientific Reports

Brendon Stubbs PhD [1-3*^], Nicola Veronese MD[4^], Davy Vancampfort PhD[5-6], A. Mathew Prina PhD[2], Pao-Yen Lin MD[7-8], Ping-Tao Tseng MD[9], Evangelos Evangelou MD[10], Marco Solmi MD[4, 11-12], Cristiano Kohler PhD [13], André F. Carvalho MD[4, 13], Ai Koyanagi MD[14-15]

^ = Joint first authors

1. Physiotherapy Department, South London and Maudsley NHS Foundation Trust, Denmark Hill, London SE5 8AZ, United Kingdom
2. Health Service and Population Research Department, Institute of Psychiatry, Psychology and Neuroscience, King's College London, De Crespigny Park, London, Box SE5 8AF, United Kingdom
3. Department of Psychosis studies, Institute of Psychiatry, Psychology and Neuroscience, King's College London, De Crespigny Park, London, Box SE5 8AF, United Kingdom
4. Institute for Clinical Research and Education in Medicine, I.R.E.M., Padova, Italy
5. KU Leuven Department of Rehabilitation Sciences, Leuven, Belgium
6. KU Leuven, University Psychiatric Center KU Leuven, Leuven-Kortenberg, Belgium
7. Department of Psychiatry, Kaohsiung Chang Gung Memorial Hospital and Chang Gung University College of Medicine, Kaohsiung, Taiwan
8. Institute for Translational Research in Biomedical Sciences, Kaohsiung Chang Gung Memorial Hospital
9. Department of Psychiatry, Tsyr-Huey Mental Hospital, Kaohsiung Jen-Ai's Home, Taiwan
10. Department of Hygiene and Epidemiology, University of Ioannina Medical School, Ioannina, Greece; Department of Biostatistics and Epidemiology, Imperial College London, London, UK
11. Department of Neurosciences, University of Padova, Padova, Italy
12. Local Health Unit 17 ULSS 17, Mental Health Department, Padova, Italy

13. Department of Clinical Medicine and Translational Psychiatry Research Group, Faculty of Medicine, Federal University of Ceará, Fortaleza, CE, Brazil.
14. Research and Development Unit, Parc Sanitari Sant Joan de Déu, Universitat de Barcelona, Fundació Sant Joan de Déu, Dr. Antoni Pujadas, 42, Sant Boi de Llobregat, Barcelona 08830, Spain
15. Instituto de Salud Carlos III, Centro de Investigación Biomédica en Red de Salud Mental, CIBERSAM, Monforte de Lemos 3-5 Pabellón 11, Madrid 28029, Spain

* **Corresponding Author**: Brendon Stubbs PhD, Head, Physiotherapy Department, South London and Maudsley NHS Foundation Trust, Denmark Hill, London, United Kingdom. Tel: 0044 208 3003100, fax 00442032282702 email: brendon.stubbs@kcl.ac.uk

Appendix 1 of ethical approval committees for the World health survey

Ethical approval

Ethical approval was obtained from each of the following committees in the respective countries for the World Health Survey:

Country	Ethical committee approving the study
Bangladesh	Mitra and Associates
Bosnia Herzegovina	The Federal Public Health Institute
Brazil	Fundacao Oswaldo Cruz
Burkina Faso	Institut de Recherche en Sciences de la Santé
Chad	Faculté des Sciences de la Santé, Univ N'Djamena
China	Centre for Health Statistics Information
Comoros	Bureau Comorien de Conseil
Congo	Unité de recherche sur les systèmes de santé
Ivory Coast	Ministère de la Santé
Croatia	The Croatian National Institute of Public Health
Czech Republic	Institute of Health Information and Statistics
Dominican Republic	Centro de Estudios Sociales y Demográficos (CESDEM)
Ecuador	Fundación Ecuatoriana para la Salud y el Desarroll (FESALUD)
Estonia	Saar Poll Ltd.
Ethiopia	Department of Community Health, Jimma University
Georgia	Georgian State Medical Academy (GSMA)
Ghana	Department of Community Health, Ghana Medical Sch
Hungary	Johan Bela National Centre for Epidemiology
India	International Institute of Population Sciences
Kazakhstan	Kazakstan School of Public Health (KSPH)
Kenya	Central Bureau of Statistics
Laos	National Institute of Public Health, Ministry of Health
Latvia	The Health Promotion Center
Malawi	Centre for Social Research (CSR)
Malaysia	Public Health Institute, Ministry of Health
Mali	Cellule de Planification et de Statistique, (CPS)
Mauritania	Office Nationale de la Statistique (ONS)
Mauritius	Mauritius Instituteof Health
Mexico	Instituto Nacional de Salud Pública
Morocco	Ministère de la Santé
Myanmar	Department of Medical Research, Ministry of Health
Namibia	Ministry of Health
Nepal	ORG-MARG Nepal PVT.Ltd
Pakistan	Ministry of Health
Paraguay	Fac.de Ciencias Veterinarias, Univ. Nacional/DGEEC
Philippines	College of Medicine, University of the Philippines
Russia	Semashko Institute for Research on Social Hygiene,
Senegal	Direction Etudes, Recherche et Formation (DERF)
Slovakia	Environment,s.c., Centre of Biostatistics and Envi
South Africa	Community Agency for Social Enquiry (CASE)
Sri Lanka	Ministry of Health
Swaziland	Faculty of Health Sciences, Univ Swaziland
Tunisia	Institut National de la Santé Publique
Ukraine	Odessa State Medical University
Uruguay	Centro de Estudios de Economia y Salud (CEES)
Vietnam	Ministry of Health
Zambia	School of Humanities & Social Sciences, University of Zambia
Zimbabwe	Community Health, University of Zimbabwe

Appendix 2 Sample size by country

**Africa**	N	**Asia**
Burkina Faso	4,948	Bangladesh
Chad	4,870	China
Comoros	1,836	India
Ethiopia	5,089	Kazakhstan
Ghana	4,165	Laos
Ivory Coast	3,251	Malaysia
Kenya	4,640	Myanmar
Malawi	5,551	Nepal
Mauritania	3,902	Pakistan
Mauritius	3,968	Philippines
Morocco	5,000	Sri Lanka
Namibia	4,379	United Arab Emirates
Senegal	3,461	Vietnam
South Africa	2,629	
Tunisia	5,202	**Europe**
Zambia	4,165	Bosnia and Herzegovina
		Croatia
**Americas**	N	Czech Republic
Dominican Republic	5,027	Estonia
Mexico	38,746	Latvia
Paraguay	5,288	Russia
Uruguay	2,996	Spain
		Ukraine

Appendix 3 Association between perceived stress and smoking (outcome) by country-income level estimated by multivariable logistic regression

	Low-income countries			Middle-income countries		
	OR	95%CI	P-value	OR	95%CI	P-value
**Perceived stress**[a]	1.06	(1.04-1.08)	<0.0001	1.02	(1.00-1.04)	0.0130
**Age** (years)						
18-34	1.00			1.00		
35-59	2.74	(2.54-2.96)	<0.0001	1.24	(1.17-1.32)	<0.0001
≥60	2.85	(2.50-3.23)	<0.0001	0.65	(0.59-0.72)	<0.0001
**Sex**						
Female	1.00			1.00		
Male	6.78	(6.20-7.42)	<0.0001	7.17	(6.68-7.69)	<0.0001
**Wealth**						
Poorest	1.00			1.00		
Poorer	0.82	(0.73-0.91)	0.0002	0.88	(0.81-0.96)	0.0032
Middle	0.70	(0.62-0.79)	<0.0001	0.91	(0.83-1.00)	0.0489
Richer	0.59	(0.51-0.67)	<0.0001	0.85	(0.77-0.93)	0.0005
Richest	0.37	(0.32-0.42)	<0.0001	0.79	(0.71-0.87)	<0.0001
**Setting**						
Rural	1.00			1.00		
Urban	0.95	(0.83-1.07)	0.3891	1.20	(1.11-1.30)	<0.0001

Abbreviation: OR odds ratio; CI confidence interval
[a] The perceived stress score ranged from 2-10 with higher scores corresponding to higher levels of perceived stress.
Models are adjusted for age, sex, wealth, setting, and country.
Country-income level was based on the World Bank classification in 2003.
Low-income countries include Burkina Faso, Bangladesh, Ivory Coast, Comoros, Ethiopia, Ghana, India, Kenya, Laos, Myanmar, Mauritania, Malawi, Nepal, Pakistan, Senegal, Chad, Vietnam, Zambia.
Middle-income countries include Bosnia and Herzegovina, China, Czech Republic, Dominican Republic, Estonia, Croatia, Kazakhstan, Sri Lanka, Latvia, Morocco, Mexico, Mauritius, Malaysia, Namibia, Philippines, Paraguay, Russia, Tunisia, Ukraine, Uruguay, South Africa.
